# Mild cognitive impairment in older adults: Analysis of some factors

**DOI:** 10.1590/1980-57642020dn14-010005

**Published:** 2020

**Authors:** Maria dos Anjos Dixe, Mônica Braúna, Timóteo Camacho, Filipa Couto, João Apóstolo

**Affiliations:** 1 Centre for Innovative Care and Health Technology (ciTechCare), Polytechnic Institute of Leiria (IPLeiria), Leiria, Portugal.; 2 The Health Sciences Research Unit: Nursing, Nursing School of Coimbra, Coimbra, Portugal.

**Keywords:** neurocognitive disorders, aged, dementia, care homes, cognitive decline, transtornos neurocognitivos, idoso, demência, casas de repouso, declínio cognitivo

## Abstract

Mild cognitive decline is a feared aspect of aging associated with frailty experienced by individuals.

**Objective:**

To determine the number of elderly people with mild cognitive impairment (MCI); to determine the relationship of sociodemographic and clinical variables by group of individuals with or without MCI and to determine the relationship between MCI assessed by 6CIT and the cognitive domains assessed by the MoCA.

**Methods:**

A correlational study was conducted of 44 elderly individuals attending a day-care center or residing in a care home, with an average age of 88.9 ± 8.8 years who answered a structured interview collecting sociodemographic and clinical data.

**Results:**

The elderly living at home had higher average body mass index and number of pathologies than those living in an institution for the elderly (p < 0.01). 63.6% of the elderly did not have MCI, and no differences were found between residential settings. The comparison between 6CIT and MoCA yielded differences in the general domain and in visual, attention, abstraction and, orientation subdomains.

**Conclusion:**

Cognitive stimulation interventions should be optimized according to the residential setting at the level of comorbidities and nutrition.

## RESUMO

O declínio cognitivo leve é um aspecto temido do envelhecimento associado à fragilidade vivenciada pelos indivíduos.

**Objetivo:**

Determinar o número de idosos com comprometimento neurocognitivo leve; determinar a relação das variáveis sociodemográficas e clínicas por grupo de indivíduos com ou sem comprometimento neurocognitivo leve e determinar a relação entre o comprometimento neurocognitivo leve avaliado pelo 6CIT e os domínios cognitivos avaliados pelo MoCA.

**Métodos:**

Estudo correlacional realizado em 44 idosos de instituições de creche e residência permanente, com idade média de 88,9 ± 8,8 anos, que responderam a uma entrevista estruturada composta por dados sociodemográficos e clínicos.

**Resultados:**

Os idosos que residem em casa apresentam maior índice de massa corporal médio e número de patologias que os idosos que vivem em uma instituição para idosos (p < 0,01). 63,6% dos idosos não apresentam comprometimento neurocognitivo leve e não são encontradas diferenças ao atravessar o contexto da residência. Na comparação entre 6CIT e MoCA produz diferenças no domínio geral e subdomínios visuais, de atenção, abstração e orientação.

**Conclusão:**

É necessário otimizar as intervenções de estimulação cognitiva de acordo com o contexto de residência no nível de comorbidades e nutrição.

The prevalence of noncommunicable chronic diseases has accompanied the increase in life expectancy. In addition, chronic diseases may compromise the functional capacity of the elderly, accelerating the process of frailty syndrome.^1^ Frailty is assumed to be a dynamic process that leads to a spiral of decline in various functional domains and exacerbates the risk of geriatric syndromes.^2^ Frailty is more prevalent with increasing age and may confer high risk for adverse health outcomes, including mortality, institutionalization, falls, and hospitalization.^3^ Between a quarter and half of people over 85 are estimated to be frail.^4^

The frailty phenotype introduced by Fried et al. postulates that five indicators (weight loss, exhaustion, idle speed, low grip strength and low physical activity) are related to each other in a cycle of frailty.^5^ In an evolutionary line, the literature demonstrates that the concept of frailty has proliferated, until today, to a broader approach that includes various domains related to the aging process, such as: nutritional, psychological, cognitive and social factors.^6^ Nevertheless, frailty remains an evolving concept, lacking both a single definition and diagnostic criteria for use in clinical practice and epidemiological research.^7^^,^ 8

In this context, an integral conceptual model was recently proposed by Gobbens et al. where frailty is defined as a dynamic state that affects an individual who experiences loss in one or more domains of human functioning (physical, psychological and social), which is caused by the influence of a number of variables and increases the risk of adverse outcomes.^9^ Among frailty phenotypes, cognitive frailty has been proposed as a clinical entity with cognitive impairment related to physical causes, with potential reversibility, and as an important target for secondary intervention in early or asymptomatic dementia.^10^

Along the same lines, the ability to perform cognitive tasks will reach its maximum around 20 years of age, decreasing thereafter throughout life, namely the ability to perform memory tasks, which increases frailty. Despite these losses and perceived weakness, it is not possible to group individuals into either a dementia or normal aging process. Thus, there is a need to categorize these subjects differently, under the name mild cognitive impairment (MCI)/Mild Neurocognitive impairment, characterized as a symptomatic state that occurs pre-dementia and converts to dementia in the absence of adequate stimulation programs.^11^^,^ 12

To this end, diagnostic criteria have been established for mild cognitive impairment that address concerns with the individual´s cognition changes noted by a caregiver or specialized professional, where objective evidence of cognitive impairment (e.g., memory, executive functions, attention, among others) is obtained through cognitive tests, the preservation of functional capacities and absence of impairment in social and occupational functioning.^11^^,^ 12

Some recommendations in clinical practice for people with frailty, which includes subjects with mild cognitive impairment, have been suggested, most notably, with strong evidence, physical exercise, nutrition and other intervention combinations, such as cognitive training.^2^ Subsequent studies revealed that physical and cognitive factors are crucial in predicting risk of death.^13^^,^ 14 Although a relationship between physical frailty and cognitive function has been reported, the severity of cognitive impairment has not been studied in relation to key aspects of physical frailty.^14^

Institutionalized older people tend to have a different health profile than older people in the community conferring a higher risk of frailty.^15^^,^ 16 Residential care facilities for older people are a new challenge, as the aging-related changes coupled with pre-existing illnesses aggravated by lack of common motivation and encouragement in this environment can make older people vulnerable to frailty and functional decline.^17^^,^ 18

The objectives of this study were to determine the number of older people with mild cognitive impairment (MCI) residing in a care facility for the elderly and at home; to determine the relationship of the variables age, number of medications, number of pathologies, dependency index, BMI and number of depressive symptoms by group of individuals according to the presence or absence of MCI and to determine the relationship between MCI assessed by 6CIT and the Cognitive Domains evaluated by the MoCA.

## METHODS

### Population and sample

This correlational study involved elderly from two institutions: a day-care facility and residential care home in the Center of Portugal.

Inclusion criteria were: i) elderly aged 65 years or older; ii) able to consent to their participation in the study; iii) with clinical conditions that allow them to participate in the study (evaluated by the institution clinician where the data were collected; iv) no previous diagnosis of cognitive impairment (assessed using the 6CIT screening scale).

Fifty-five potential participants, identiﬁed by the institution clinician of each elderly end-user organization, were screened for eligibility. Of these, eleven individuals did not meet the inclusion criteria. After applying these criteria, the sample consisted of 44 elderly and data were collected in May 2018.

The 44 participants were evaluated by an advanced trained neuropsychologist with protocol knowledge and each interview lasted about 30 minutes.

### Instruments

The instruments were applied through a face-to-face interview by a psychologist trained in neurocognitive rehabilitation:

- Sociodemographic and clinical characteristics: age, gender, marital status, academic qualifications, visual impairment, hearing impairment, degree of dependence (Barthel index),^19^ body mass index, medication intake, number of medications and place of residence.
- The Six-Item Cognitive Impairment Test (6CIT). This instrument is a cognitive screening test consisting of six simple questions that provide data on space and time orientation, attention and working memory, and delayed recall. The application time of this test is very short, not exceeding 5 minutes. Scoring ranges from 0 to 28 points indicating the absence and maximum number of errors, respectively.^20^ The proposed cutoff operating values for the Portuguese population, from which the presence of cognitive impairment is distinguished, are as follows: ≥ 16 for zero to two years of education, ≥ 11 for three to six years of education and ≥ 9 for seven years or more of education.
- Geriatric Depression Scale (GDS) 10. This instrument assesses the presence of characteristic depressive symptoms of old age. It is a hetero-rating scale, where each question is rated with 0 or 1 point. GDS10 items 2,3,6,8 and 10 are rated with 0 points in the absence of the symptom (no answer), and 1 point when it is present (yes answer). The remaining items have reverse quotation. The cut-off point was a total value of 2, establishing a division between individuals with or without symptoms suggestive of depressive disorder.^21^
- Montreal Cognitive Assessment (MoCA). This instrument evaluates eight cognitive domains comprising several tasks in each domain: attention and concentration, executive functions, memory, language, visuo-constructive abilities, capacity for abstraction, calculation and orientation. The total score is 30 points, with a score of 26 or higher considered normal.^22^

### Data processing

To systematize and highlight the information provided by these same data, we used descriptive statistics techniques: absolute and relative frequencies, measures of central tendency, and measures of dispersion and variability using the Statistical Package for the Social Sciences (SPSS) version 24.0.

The Mann-Whitney test, the alternative t-test for two independent samples and the Chi-square independence test were used to analyze the relationship between two qualitative variables. Nonparametric tests were used based on the typology of the variables: non-normal distribution and sample size (< 30 precluding the use of Central Limit Theorem). In all situations the significance level adopted was p ≤ 0.05.

### Formal and ethical procedures

This study is part of a larger project entitled Mind & Gait and was approved by the National Data Protection Commission (No. 11802) and the Ethics Committee of the Coimbra Higher School of Nursing (No. P455-09 / 2017). It was also approved by the institutions where the study took place.

## RESULTS

### a) Characterization of the sociodemographic, academic and health variables of the sample

Of the 44 elderly who participated in the study, 20 attended a day-care facility and 24 lived in a residential care home for the elderly. The sample had an average age of 80.9 ± 8.8 years and predominance of females (34.1%). About 97.7% took an average of 7.4 ± 2.9 medicines per day. The sociodemographic, academic and health characteristics for the total sample and by place of residence are shown in Table 1. Regarding the dependence index (U = 198,000; p > 0.05) and depressive symptoms (U = 220,500; p > 0.05), there were no statistically significant differences between participants regarding their place of residence. Regarding BMI and pathologies, the elderly in the home setting had higher values (32.1 ± 5.8; 4.5 ± 2.1) when compared to residents in Residential Care Homes for the elderly (25.8 ± 4.6; 3.8 ± 2.1), with the differences being statistically significant. The results described below show: BMI (U = 96,000; p = 0.001); and number of pathologies (U = 154,000; P = 0.039).

**Table 1:** Sample distribution regarding academic and health sociodemographic data.

Academic and health sociodemographic variables		Total sample			Residents at ERPI			Home Residents	
		Nº	%		Nº	%		Nº	%
Sex	Male	15	34.1		10	41.7		5	25.0
	Female	29	65.9		14	58.3		15	75.0
Academic qualifications	0 years	8	18.2		3	12.5		5	25.0
	1 to 4 years	28	63.6		16	66.7		12	60.0
	5 to 6 years	3	6.8		2	8.3		1	5.0
	7 to 9 years	2	4.5		2	8.3		1	5.0
	10-12 years	1	2.3						
	Higher Education	2	4.5		1	4.2		1	5.0
Visual deficit	Yes	35	79.5		17	70.8		18	90.0
	Not	9	20.5		7	29.2		2	10.0
Hearing deficit	Yes	17	39.5		6	25.0		11	57.9
	Not	26	60.5		18	75.0		8	42.1
Take medication	Yes	43	97.7		24	100.0		19	95.0
	Not	1	2.3		0	0		1	5.0

### b) Relationship between Mild Cognitive Impairment and Cognitive Domains

Analysis of the results of the 6 CIT according to the cutoff values revealed that 28 (63.6%) of the elderly (15 residing in the Residential Care Homes for the elderly and 13 at home) had values below the reference values, as opposed to 16 (36.4 %) (of which 9 were care home residents and 7 at home). Application of the appropriate test for the type of variables showed that the differences do not have statistical significance when stratifying the elderly by place of residence (χ^2^ = 0.000; p > 0.05).

### c) Relationship between age, number of medications, number of diseases, dependence index, BMI and number of depressive symptoms by group of individuals according to the presence or absence of MCI

Based on the non-existence of differences between the 6 CIT values by place of residence, we chose not to separate the groups of elderly people living in Residential Care Homes for the elderly and at home in the following relationships. Analysis of the differences between the 6 CIT values for variables with reference only to the presence or absence of MCI (Table 2) revealed no statistically significant differences between the absence and presence of MCI in relation to age, number of drugs used, degree of dependence, number of depressive symptoms and BMI (p > 0.05).

**Table 2:** Results of the application of the Mann Whitney U test in the variables age, number of medications, number of diseases, dependence index, BMI and number of depressive symptoms by group of individuals according to the presence or absence of MDI.

6CIT Prevalence		Mean Rank	Average	Median	Sd	U	P
Age	< vr	21.23	79.6	80.5	8.25	188,500	0.386
	≥ vr	24.72	82.18	82.5	8.87		
Number of medicines	< vr	23.79	7.25	7	3.44	188.000	0.378
	≥ vr	20.25	6.25	6	3.15		
Barthel	< vr	22.88	77.32	80	12.65	213.500	0.796
	≥ vr	21.84	75.63	77.5	14.24		
GDS-10	< vr	22.11	3.25	2	2.97	213.00	0.786
	≥ vr	2319	3.25	3	2.51		
Number of pathologies	< vr	23.59	4.07	4.00	2.20	193.500	0.449
	≥ vr	20.59	3.50	3.50	2.03		
IMC	< vr	22.39	28.86	26.94	6.74	221.000	0.942
	≥ vr	22.69	28.41	28.83	4.65		

vr: reference values.

### d) Relationship between MCI assessed by 6CIT and Cognitive Domains assessed using MoCA

We also tested for differences regarding the presence and absence of MCI as measured by the 6-CIT versus the cognitive domains of the MoCA. Significant differences were found in the General Domain and sub domains: visual, attention, abstraction, and orientation. It is noteworthy that in dimensions where differences between groups were statistically significant, individuals with 6 CIT values lower than the reference value had higher mean and median values on these dimensions and for total score (Table 3).

**Table 3:** Results of the application of the Mann Whitney U test in the Cognitive Assessment variable and its dimensions by group of individuals with and without MDI.

Prevalence_6_cit		Mean Rank	Average	Median	SD	U	P
Total MoCA subdomains	<vr	26.89	17.32	18	4.119	101	0.003
	≥vr	14.81	12.63	12.5	4.559		
Visual	<vr	26.27	2.46	2	1.503	118.5	0.008
	≥vr	15.91	1.25	1	0.856		
Appointment	<vr	21.64	2.21	2	0.738	200	0.522
	≥vr	24	2.38	2	0.619		
Attention	<vr	25.68	3.29	3	1.823	135	0.028
	≥vr	16.94	2.06	1.5	1.692		
Language	<vr	24.29	1.39	1	0.956	174	0.196
	≥vr	19.38	1	1	0.73		
Abstraction	<vr	26	1.07	1	0.663	126	0.01
	≥vr	16.38	0.5	0	0.73		
Evocation	<vr	24.86	1.61	2	1.474	158	0.086
	≥vr	18.38	0.81	0	1.377		
Guidance	<vr	25.55	5.39	6	0.786	138.5	0.025
	≥vr	17.16	4.63	5	1.31		

vr: reference values.

## DISCUSSION

Forty-four elderly people participated in this study, 24 of them living in a Residential Care Home for the elderly and 20 at home while attending a Day-care Center. The sample comprised mostly female elderly, with 1 to 4 years of education, visual impairment and in use of medication.

There are few studies comparing elderly residents in Residential care facilities for the elderly and in the community. Cognition is considered a component of frailty and has been shown to be associated with adverse health outcomes.^23^^,^ 24 In the context of institutionalization, the elderly are in an evident situation of physical, cognitive and emotional frailty. Studies have shown that mean score on the Mini-Mental State Examination (MMSE) is lower among the frail and negatively impacts the health of these elderly and may accelerate the process of frailty.^25^ Level of education may contribute to the formation of cognitive reserve and justify the high frequency of MCI among the elderly analyzed. In the FIBRA study, education was the variable that best explained the variability in Mini-Mental State Exam total score.^26^ Similar results have been explained by many authors through the cognitive reserve model.^25^^,^ 27 This model suggests that exposure to education and complex life-long activities favors the preservation of cognitive ability and resistance to neural damage.

The impact of various nutritional factors on physical frailty and its components has been of recent interest^28^ and current epidemiological evidence also supports the hypothesis that diet-related factors may be associated with cognitive decline in old age. More sedentary lifestyle, reduced metabolic mass and, consequently, lower energy expenditure and food consumption, are important contributors to the progression of frailty. Malnutrition may be a major cause of frailty, and elderly with protein-energy malnutrition, a treatable condition, tend to have poor cognitive performance.

In this study, we found that the elderly residents of the Residential Care Homes for the elderly had a better BMI than those who lived at home, probably due to greater dietary rigor, however, there were no statistically significant differences between the BMI value and the presence or absence of MCI. From a pathophysiological perspective, the etiology of the cognitive-frailty association seems to be multifactorial and several mediators or possible pathways have been suggested to explain these. Hormonal and inflammatory links and processes, along with nutritional, vascular, neuropathological and metabolic influences may be of great relevance.^10^

Although the results of this study did not reveal a strong influence of the number of depressive symptoms on cognitive impairment, studies indicate that depression is a risk factor and consequence of frailty, and affects cognitive function. This suggests that a mechanism underlying the link between frailty and cognition may involve psychological factors such as mood disorders.^29^ The relationship between level of dependence and cognitive impairment also showed no significant effects. However, other studies reveal an association between cognition and functioning, identifying a proportion of elderly with cognitive decline dependent on basic activities of daily living.^30^^,^ 31 The performance of activities of daily living in the institutional context is more limited, given that the environment, organization, norms and routines directly impact decision-making and execution of these tasks. Results from a cross-sectional study identified that physical frailty, cognitive impairment and cognitive frailty have an impact on the performance of activities of daily living,^32^ reinforcing that elderly people with comorbid frailty and cognitive impairment had an increased risk of limitations in these activities compared to healthy elderly or elderly with cognitive impairment or frailty.

In the present study, the number of pathologies and number of medications was also uncorrelated with the presence or absence of MCI. The fact that we did not control for medication typology could be an important factor for these results.

A strong relationship was also detected between having MCI and changes on all cognitive domains assessed by the MoCa, as reported in other studies.^33^

There are several limitations of this study, namely the size of the sample, the fact that the elderly living in the home attended a care center during the day and performed several activities that were the same as the group residing in the Residential Care Homes for the elderly. There was also recruitment of older people living in the home only. There is still a need to conduct randomized intervention studies investigating the role of nutrition and/or exercise together with a cognitive stimulation program in cognitively frail individuals with possible progression to dementia.

In conclusion, the elderly who lived at home had a higher average body mass index and more pathologies than the elderly living in an institution for the elderly. More than half of the elderly had no mild cognitive impairment, and no differences were found between residential settings. The comparison between the 6CIT and MoCA yielded differences in the general domain and visual, attention, abstraction and, orientation subdomains.
